# What every intensivist should know about the management of peritonitis in the intensive care unit

**DOI:** 10.5935/0103-507X.20180007

**Published:** 2018

**Authors:** Jan J. De Waele

**Affiliations:** 1 Department of Critical Care Medicine, Ghent University Hospital - Ghent, Belgium.

## Introduction

Complicated intra-abdominal infections (cIAI) are among the more challenging infections in the intensive care unit, not only because they are typically associated with more severe organ dysfunction^(1)^ but also because the need for an intervention is more important than in other infections.^(2)^ In recent years, a number of new challenges have been added, further complicating the decision-making process for these patients, who are often vulnerable. In this document, we will focus on the key elements in the management of these patients and review the latest evidence.

## Multidrug resistance is a pressing issue, also in peritonitis...

Multidrug resistance (MDR) is increasing worldwide, and there are important geographical differences for various pathogens.^(3)^ Whereas most of the data on MDR focus on respiratory tract infections, it is a similar issue in cIAI, most notably in patients with hospital acquired infections.^(4,5)^

There are a number of reasons why MDR is a highly relevant topic in cIAI. First, cIAI are typically polymicrobial infections, and resistance may be present in both Gram-negative (most notably *Enterobacteraceae*) and Gram-positive pathogens, such as *Enterococcus faecium,* in the same patient. Second, the duration of antibiotic therapy is often longer and driven by a lack of a source of control, requiring multiple surgical interventions.^(6,7)^ This is most explicitly reflected in patients with tertiary peritonitis, for whom often-resistant pathogens that are difficult to eradicate cause persistent inflammation of the peritoneal cavity.^(8)^

Multidrug resistance is particularly problematic in hospitalized patients and in patients who have been exposed to antibiotics prior to or for the therapy of the current cIAI.^(9,10)^ However, MDR is also increasing in community-acquired infections, with extended-spectrum beta-lactamase (ESBL)-producing bacteria being the most globally widespread problem, although there is important regional variation in its prevalence.^(11,12)^ In nosocomial infections,^(4,9,13)^ both Gram-positive and Gram-negative pathogens that have MDR may be encountered, and these include methicillin-resistant *Staphylococcus aureus* (MRSA), vancomycin-resistant enterococci (VRE) and ESBL-producing *Enterobacteraceae* but also carbapenem-resistant *Enterobacteraceae* (CRE), MDR *Pseudomonas aeruginosa* and *Acinetobacter* spp., which also have important geographical variations.

## ..., which makes rational antibiotic use even more important today

The threat of MDR inevitably poses problems in the empirical therapy of cIAI, and the use of multiple and broad-spectrum antibiotics is a logical consequence that fuels the vicious cycle.^(3)^

Empirical antibiotic therapy should therefore be based on knowledge of the local epidemiology, and the antibiotic with the least broad spectrum is preferred.^(14)^ Patients at the highest risk of MDR infection include those who have been treated with antibiotics and patients who have been residing in the hospital in the previous weeks. Because of the same problem of MDR, intraoperative cultures are mandatory in nosocomial infections^(15)^ and antibiotic de-escalation can be safely performed when cultures are available.^(6)^

However, the first question should be whether the patient really needs antibiotics. There are a number of conditions often encountered in the intensive care unit (ICU) that do not need antibiotic therapy beyond the prophylactic antibiotics related to a surgical intervention (Table 1).^(14)^ Particularly relevant to the ICU are postoperative patients who had abdominal trauma from intestinal ischemia that required intervention. Another source of concern in patients who have been treated for abdominal infections are abdominal drains. Cultures from abdominal drains should be carefully interpreted, as biofilms and colonization occur very frequently. As it is often difficult to discriminate infection from colonization, routine cultures of abdominal drains are not recommended. Although these can identify recurrent infections or an inadequate source control, their actual value in the management of abdominal infections has not been demonstrated.

**Table 1:** Conditions for which therapeutic antimicrobials (> 24 hours) are not recommended, assuming that the source has been adequately controlled*

Traumatic or iatrogenic enteric perforations operated on within 12 hours
Gastroduodenal perforations operated on within 24 hours
Acute or gangrenous appendicitis without perforation
Acute or gangrenous cholecystitis without perforation
Transmural bowel necrosis without perforation, established peritonitis, or abscess

* Source: Based on: Mazuski JE, Tessier JM, May AK, Sawyer RG, Nadler EP, Rosengart MR, et al. The Surgical Infection Society Revised Guidelines on the Management of Intra Abdominal Infection. Surg Infect (Larchmt). 2017;18(1):1-76.^(14)^

## Pharmacokinetic properties of antibiotics have been insufficiently studied in complicated intra-abdominal infections patients

There is ample evidence suggesting that the pharmacokinetics of antibiotics in critically ill patients is different from that in healthy volunteers or patients in the general ward.^(16)^ This is mainly driven by increases in the volume of distribution and changes in the elimination of the drug. Most of the antibiotics used in the treatment of peritonitis are beta-lactam antibiotics, which are eliminated from the circulation by the kidneys. Therefore, their elimination can be enhanced (e.g., in patients with augmented renal clearance) or reduced (e.g., in patients with acute kidney injury (AKI)).

These pharmacokinetic (PK) changes are enhanced in peritonitis by a number of additional challenges.^(17)^ First, these patients often require surgery, in which blood loss and volume replacement may further reduce the antibiotic concentrations. Also, bleeding from the surgical site may persist into the postoperative period and affect the PK status. Moreover, loss of fluids, those that are often protein-rich, may further lead to losses of antibiotics via the abdominal drains. In patients treated with open abdomen therapy in the early postoperative phase, active negative pressure may induce significant fluid losses via the abdominal cavity.

As a result, antibiotic concentrations in the peritoneal fluid at the site of infection are highly unpredictable, and this has been poorly investigated.

## New antibiotic agents are available for multidrug-resistant pathogens but should be used selectively

Several new antibiotics that include MDR pathogens in their spectrum have been introduced recently, and most of them have been studied in cIAI.^(18)^ Although there are some concerns that critically ill patients were often excluded from these studies, it can be assumed that new antibiotics or antibiotic combinations such as ceftolozane/tazobactam, ceftazidime/avibactam, and eravacycline can be successfully used in ICU patients with cIAI^(19-21)^ (Table 2). The cephalosporin-based drugs need to be combined with metronidazole to adequately cover the anaerobic pathogens. Given the number of new drugs coming to the market, these need to be used only in patients with a high risk of MDR involvement or in directed therapy.^(12,22)^

**Table 2:** Recently introduced antibiotics for complicated intra-abdominal infections and their recommended dosings

Antibiotic	Antibiotic class	Dose	Dosing frequency	Oral formulation available	Antimicrobial spectrum highlights	Remarks
Ceftolozane/tazobactam	Cephalosporin + betalactamase inhibitor	1,000mg/500mg	3 times/day	No	Selected ESBL-producing *Enterobacteraceae* and MDR *Pseudomonas aeruginosa*, but not KPC	Combined with metronidazole for anaerobe coverage
Ceftazidime/avibactam	Cephalosporin + betalactamase inhibitor	2,000mg/500mg	3 times/day	No	Selected ESBL-producing *Enterobacteraceae* and CRE, including KPC	Combined with metronidazole for anaerobe coverageClinical cure rate in a phase 3 study was lower in patients with renal impairment
Eravacycline	Fluorocycline	1mg/kg	2 times/day	Yes	MRSA, VRE, ESBL *Enterobacteraceae* and CRE, but not *Pseudomonas aeruginosa*	

ESBL - extended spectrum beta-lactamase; MDR - multidrug resistance; KPC - Klebsiella Pneumoniae Carbapenemase; CRE - carbapenem-resistant *Enterobacteraceae*; MRSA - methicillin resistant *Staphylococcus aureus*; VRE - vancomycin-resistant enterococci.

## Intra-abdominal candidiasis is a severe infection for which adequate early therapy is important, but antifungal prophylaxis for all patients with peritonitis does not appear to be beneficial

Fungal involvement in cIAI is common, particularly in nosocomial infections and in patients who have been exposed to antibiotic therapy previously.^(23)^ Intra-abdominal candidiasis has been linked with a high mortality, and its delayed administration is a risk factor for mortality (among other factors, such as the severity of illness).^(24,25)^ Although regional variation may be present, *Candida albicans* is still the most commonly found pathogen.^(25,26)^ For other infections, source control is a critical element in intra-abdominal candidiasis,^(26)^ and when it is absent, adequate antifungal therapy has no relevant impact.

Intra-abdominal candidiasis or suspicion thereof is an important trigger for starting antifungal therapy in critically ill surgical patients. Many attempts to identify patients at risk of intra-abdominal candidiasis have been made in the past. Frequently used tools to predict the involvement of candida in ICU infections include the Colonization Index,^(27)^ Candida score,^(28,29)^ and Clinical Prediction Rule. Although these tools performed well in their original patient cohorts, their external validity seems to be limited, and their usefulness in clinical practice seems to be minimal.^(30)^ For the prediction of candida involvement in abdominal infections, Dupont et al. developed a score that includes the length of stay before surgery, preoperative cardiovascular failure, generalized peritonitis, and upper gastrointestinal tract perforation as the relevant risk factors.^(31)^ The usefulness of this model needs to be confirmed.

In recent years, more studies trying to identify the role of antifungal therapy have been performed. A large RCT comparing micafungin with placebo in patients requiring surgery and who were admitted to the ICU could not demonstrate any advantage of antifungal treatment in this setting, albeit that the antifungal therapy may have been administered too late to be effective.^(32)^

Currently, antifungal prophylaxis can be considered in patients with anastomotic leakage after abdominal surgery based on smaller (and older) studies.^(33)^ When the presence of *Candida* spp. is confirmed, adequate therapy is imperative, and an echinocandin is the preferred choice for critically ill patients.^(34)^ When possible, de-escalation to azoles can be considered in patients who are clinically improving.

## Source control is a pivotal component of complicated intra-abdominal infections treatment

In patients with sepsis and septic shock, antibiotic therapy has been studied intensively and is consistently considered as being critical in the management of severe infections.^(35)^ Although antibiotics are indeed essential, source control is at least as important as antibiotics and is highly relevant in patients with abdominal infections. However, it has been studied quite poorly, is hard to quantify, and requires the help of other specialties, such as surgery and interventional radiology. Source control focuses on the elimination of a septic focus and the control of ongoing contamination, and in the context of a cIAI, it typically requires a surgical intervention or percutaneous drainage (PCD). A lack of source control is a particular problem and inevitably contributes to prolonged antibiotic therapy, the selection of resistant pathogens and tertiary peritonitis. Recent data show that source control timing is equally important, and in critically ill patients, Bloos et al. found that a delay beyond 6 hours was associated with a 2.3-fold increase in mortality. In fact, source control was the only modifiable risk factor present.^(36)^ Percutaneous drainage is now increasingly used and avoids the risk of additional injury during a surgical intervention, although not all patients or infections are suited for treatment by PCD.^(37)^ If there is ongoing contamination, multiple abscesses, thick necrosis or diffuse peritonitis, PCD may be of limited value. Imaging plays a critical role here and will help to appropriately select patients for either intervention. There are multiple obstacles to early source control, including a lack of clinical symptoms, concomitant infections, the perceived need for more investigations and availability of radiology, including that of ultrasound.^(35)^ Institutional issues, such as problematic access to the operating theater, may also be present.

## Less is more in antibiotic therapy when it comes to duration of therapy

The duration of antibiotic therapy is generally not one of the priorities in managing patients with cIAI. In most reports, the therapy continues for 10 - 14 days, with typically longer courses in nosocomial infections. Uncertainty in source control and the inappropriate interpretation of abdominal drain cultures certainly contribute to this phenomenon. However, it has been demonstrated that continued antibiotic therapy does not prevent treatment failure in patients with cIAI,^(38)^ and a recent randomized controlled trial (RCT) from France found similar outcomes in patients with postoperative peritonitis who were treated for 8 days or 15 days (Montravers P, personal communication). In 2015, a multicenter RCT from the USA (the STOP-IT study) found that patients with peritonitis and an adequate source control can be treated with 4 days of antibiotic therapy. This was a well-designed study, although it should be mentioned that most of the patients were not critically ill, and extrapolating data to the ICU should be done cautiously.^(39)^ Subgroup analyses on patients with sepsis did not find any advantage of prolonging therapy beyond 4 days, but the numbers were small, and the study was not powered to detect any effects on the major endpoints.^(40)^ The updated guidelines from the Surgical Infection Society recommend limiting antimicrobials to 96 hours in patients who have had adequate source control and limiting it to 5-7 days in patients for whom a definitive source control procedure was not performed.^(14)^ In a subgroup of patients with cIAI included in the SAPS study, procalcitonin (PCT)-guided antibiotic duration was not different from the standard therapy.^(41)^ Apparently, cIAI are different from other infections when it comes to the role of PCT in guiding antibiotic therapy.

## Temporary abdominal closure is increasingly used in the context of peritonitis

In recent years, there has been an increase in the use of open abdomen therapy (OAT), mainly in the management of trauma patients to prevent or treat abdominal compartment syndrome.^(42)^ However, the concepts of damage control in the trauma setting are now also being applied in patients with severe peritonitis. This includes a rapid intervention aimed at removing the focus of infection and associated necrosis, temporarily controlling ongoing contamination (e.g., using staplers to seal perforations), and planning for secondary repair after restoration of the physiology of the patient. As in trauma, these patients are at increased risk for intra-abdominal hypertension due to an increased intra-abdominal volume because of ileus obstruction, ischemia/reperfusion injury, and accumulation of resuscitation fluids and due to decreased abdominal compliance because of pain and the laparotomy incision. Hence, OAT is increasingly applied as a preventive strategy. When an OAT approach is used, negative pressure wound therapy with fascial traction is reportedly the best solution in terms of complications and closure rates.^(43)^ Techniques such as the Bogota bag, mesh techniques and Zipper are associated with a significant risk for fistula and tend to have low abdominal closure rates.

Studies have also been investigating the potential effect of negative pressure wound therapy (NPWT) on removing inflammatory cytokines from the abdominal cavity. Indeed, in an animal model, systemic inflammation was attenuated by removing the abdominal fluid using NPWT, which had a beneficial effect on organ function.^(44)^ In the only human study so far, Kirkpatrick et al. observed a reduced mortality in NPWT but could not demonstrate any effect on systemic inflammation,^(45)^ and the exact role of NPWT needs to be elucidated.

## Conclusion

Managing complicated intra-abdominal infections in the intensive care unit remains a challenging issue, with an increase in multidrug resistance infections being the most imminent threat to patients with peritonitis, which further stresses the role of rational antibiotic use for both older and newly introduced antibiotics, such as ceftolozane/tazobactam, ceftazidime/avibactam and eravacycline, which should be used preferably in patients with documented multidrug resistance infections or in those who are at an increased risk of multidrug resistance involvement. In this context, there has been an increased interest in the pharmacokinetic/pharmacodynamic of antibiotics in abdominal infections, but many questions remain. The duration of antibiotic therapy should be carefully considered, but evidence is accumulating that shorter courses are equally as effective provided that the infection is controlled. Apart from antibiotics, source control is pivotal for a successful outcome. Percutaneous drainage is a valuable alternative to surgical intervention, but its exact role remains to be determined. Temporary abdominal closure is a promising technique but should be used selectively until more evidence becomes available.
